# Causation and Treatment Algorithms for Elderly Patients who have Fallen in the Twin Tiers

**DOI:** 10.7759/cureus.6513

**Published:** 2019-12-30

**Authors:** Alicia J Harbison, Sheela Prabhu

**Affiliations:** 1 Family Medicine, Transcend Medical Group, Pantego, USA; 2 Internal Medicine, Guthrie Robert Packer Hospital, Sayre, USA

**Keywords:** geriatrics, falls, vitamin d, physical therapy, polypharmacy, family medicine, internal medicine

## Abstract

Introduction

One-third of people aged 65 years of age and older fall annually.^ ^Vitamin D is key in maintaining muscle mass and bone structure. The purpose of this quality improvement project is to evaluate the current treatment of elderly patients who experience falls, educate providers on the importance of vitamin D, and measure the changes.

Methods

We obtained baseline data from the electronic medical records (EMR) of patients who had experienced falls from over a two-year period. We also surveyed providers from the departments of internal medicine (IM) and family medicine (FM) to evaluate fall treatment, educated providers on new protocols, reviewed EMRs of patients that had fallen and surveyed FM and IM providers after education.

Results

We found that vitamin D supplementation and home health (HH) referral were marginally improved after education and that significant improvement was found in patients being referred to physical therapy (PT).

Conclusion

Establishing a fall treatment protocol leads to more consistent care among FM and IM providers. Reviewing and updating of the protocol based on outcomes and subsequent research is recommended for improvement in patient care.

## Introduction

Vitamin D is a fat-soluble vitamin that is converted in the liver to 25(OH)D (circulating form) and then in the kidney to 1,25-dihydroxyvitamin D (active form). Vitamin D helps with enterocyte differentiation, intestinal absorption of calcium, intestinal phosphate absorption, direct suppression of parathyroid hormone, regulation of osteoblast (bone-forming cells) function, parathyroid hormone (PTH)-induced osteoclast (bone-resorbing cells) activation, and bone resorption [1]. Osteoporosis is characterized by low bone mass, microarchitectural disruption, and increased skeletal fragility. The best treatment and prevention of osteoporosis is adequate nutrition of calcium (Ca) and vitamin D [2].

One-third of people 65 years of age and older fall each year and many falls may result in a serious injury [3]. Sarcopenia (low muscle mass) is associated with an increased risk of disability, falls, and mortality. Vitamin D deficiency may contribute to sarcopenia [4]. In turn, falls may cause lower 25(OH)D concentrations from trauma, hospitalization, loss of independence and institutionalization that can result in a decrease in exercise to the muscles and insufficient exposure to the sunlight [5].

There are three main ways to raise vitamin D levels: ingesting food with high D levels (milk, cod liver oil), exposure to the sunlight, or taking supplements. Current dietary recommendations are 1200 mg of Ca and 800 international units (IU) of vitamin D for postmenopausal women with osteoporosis. For all other adults, the dietary recommendations are 1000 mg of Ca and 600 IU of vitamin D [2]. Since most adults are unable to accrue enough vitamin D from meals and sunlight exposure alone, it is recommended that they take supplements. There are two main forms of vitamin D supplements: ergocalciferol (D2) and cholecalciferol (D3), with studies showing that D3 increases serum 25(OH)D more effectively. Side effects of too much vitamin D include hypercalcemia, hypercalciuria, kidney stones, and the increased risk of cancers, mortality, and falls [2].

It has been recommended to have a minimum level of 25(OH)D concentrations of 30 ng/ml to minimize the risk of falls and fractures [1]. A fall has been defined as, “an event report either by the faller or a witness, resulting in a person inadvertently coming to rest on the ground or another lower level, with or without loss of consciousness or injury” [6]. Muscle weakness is seen when vitamin D levels dip to <10 or <20 ng/ml in older adults [7].

Multiple studies agree that supplementation with vitamin D is worthwhile to decrease the risk of falls and fractures, but the dosage requirements are inconsistent. For example, in a study by Zheng et al., there was an increased risk of falls for patients treated with high dosage and intermittent vtamin D when compared with the control (p = 0.006) [8], which indicated that this supplementation was ineffective in preventing mortality as it probably could not provide adequate concentrations in the blood over time; moreover, no benefit was seen in fracture and fall prevention [8]. In a study by Girgis et al., compared with the placebo, higher doses of vitamin D (700-1000 IU) reduced falls risk by 19%, but lower doses had no effect [4]. Reduction in falls was most prominent in patients who were deficient at baseline with Ca co-administration [4]. In a study by Sanders et al., participants receiving annual high-dose oral D3 experienced 15% more falls and 26% more fractures than the placebo group [6]. The increased likelihood of falls in the vitamin D group was exacerbated over a three-month period immediately after the annual dose [6]. For another study, they saw that there was no association between falls and serum vitamin D intake levels [3]. Additionally, in a study by Annweiler and Beauchet, lower levels of 25(OH)D (<10 ng/ml) corresponded with a higher risk of falls [5].

The American Geriatrics Society (AGS) has attempted to clear some confusion for the vitamin D debate. AGS noted that serum concentrations of 25(OH)D <30 ng/ml have been associated with balance problems, impaired lower extremity function, high fall rates, low bone mineral density, and muscle weakness. In order to treat this issue, clinicians should recommend vitamin D supplementation of at least 1000 IU as well as Ca supplementation to community-dwelling older adults (≥65 years old) to reduce the risk of fractures and falls. Routine lab testing for 25(OH)D serum concentrations before supplementation is not necessary. If clinicians choose to monitor 25(OH)D, they are advised to test after four months of D3 supplementation to confirm that appropriate levels have been achieved. Clinicians should not recommend large bolus doses of D2 or D3 ≥300,000 IU [9]. 

## Materials and methods

Despite the confusion of how and when to prescribe vitamin D, it was determined that examining how supplementation is utilized among the providers of internal medicine (IM) and family medicine (FM) was beneficial to standardize treatment. Our objectives were: (1) to review patient records of those who were at risk of falling, specifically vitamin D levels, vitamin D supplementation, referrals to therapy and frequency of falls; (2) prospectively survey providers to evaluate barriers to vitamin D supplementation and therapy in individuals at risk for falls; (3) establish a recommended patient care protocol and educate providers; (4) conduct a post-education review of records to see if therapy referrals and vitamin D supplementation are aligned with the patient care protocol.

Part 1

A retrospective chart review was performed to see how providers treated those who had fallen. Study participants were selected from Robert Packer Hospital (RPH), a 254-bed tertiary care teaching hospital in Sayre, Pennsylvania that serves the region of the Twin Tiers, the Northern Tier of Pennsylvania and the Southern Tier of New York State. RPH is part of the Guthrie Clinic (GC), a not-for-profit, integrated health care organization. A list of patients was selected whose providers had used a variant of the ICD-10 code for fall for their GC visit of FM or IM providers, from the period of January 8, 2016 to November 29, 2017. The patients were stratified according to demographics, body mass index (BMI), vitamin D and Ca levels, polypharmacy (defined as more than four medications), treatment chosen (supplementation and/or referral to therapy), and the cause of fall. The data was then analyzed for trends.

Part 2

A survey was designed and administered to providers from IM and FM, to evaluate how they treat fall victims and to understand possible barriers to implementing vitamin D supplementation and other treatment options, including referrals to therapy (Supplementary Text S1). Survey results were then combined and analyzed. The results of the survey along with the research done to date and education from an interdisciplinary team of professionals that treat fall victims were used to develop a patient care protocol for treatment which was then communicated to providers at grand rounds.

Part 3

Twelve months after grand rounds, a follow-up review of patient records who had fallen and a second survey of IM and FM providers was conducted to determine how well physical therapy referrals and vitamin D supplementation dosages aligned with the established protocol (Supplementary Text S2).

Statistical analysis was completed using R statistical software program (R Core Team [2014], R Foundation for Statistical Computing, Vienna, Austria). In the paper, statistical significance was defined as a p-value of 0.05 or less.

## Results

Part 1

General Characteristics

A total of 243 unique patient charts were examined who had fallen over the past two years, 65% were female, 35% were male. The average patient age was 79 years old. The average BMI was 29.66 (standard deviation=6.95), with frailty mentioned in only 3% of patients. The most common cause of falls was described as mechanical (73%). Physical therapy (PT) was recommended 33% of the time, and home health (HH) was recommended 12% of the time. Polypharmacy was found in 89% of the patients (Table 1).

**Table 1:** Comparison of pre-intervention vs. post-intervention data pulled from EMRs about elderly patients who had fallen vs: versus; n: number; CV: cardiovascular; neuro: neurological; Y: Yes; N: No; EMR: electronic medical record

Overall pre-intervention vs. post-intervention data						
		Pre-intervention (n = 243)		Post-intervention (n = 176)		p-value
		Count	Proportion	Count	Proportion	
Gender	Female	158	65.02%	128	72.73%	0.1173
	Male	85	34.98%	48	27.27%	
Cause of fall	Mechanical	177	72.84%	104	59.09%	0.0005
	Multifactorial	8	3.29%	0	0.00%	
	Drugs	6	2.47%	0	0.00%	
	CV/neuro	24	9.88%	11	6.25%	
	Risk of falls	8	3.29%	39	22.16%	
	Blind	1	0.41%	0	0.00%	
	Unknown	19	7.82%	22	12.50%	
Polypharmacy	Yes	217	89.30%	167	94.89%	0.0628
	No	26	10.70%	9	5.11%	
Low vitamin D	Yes	61	34.46%	41	29.93%	0.4656
	No	116	65.54%	96	70.07%	
Vitamin D supplements	Yes	124	51.03%	92	52.27%	0.8788
	No	119	48.97%	84	47.73%	
Vitamin D supplements if low vitamin D	Yes	35	57.38%	25	60.98%	0.8753
	No	26	42.62%	16	39.02%	
Low calcium	Yes	4	1.65%	4	2.27%	0.7253
	No	239	98.35%	172	97.73%	
Calcium supplements	Yes	55	22.63%	44	25.00%	0.6554
	No	188	77.37%	132	75.00%	
Home health	Yes	21	8.64%	13	7.43%	0.4622
	No	215	88.48%	160	91.43%	
	Declined	7	2.88%	2	1.14%	
Home health (Y/N)	Yes	28	11.52%	15	8.57%	0.4141
	No	215	88.48%	160	91.43%	
Physical therapy	Yes	63	25.93%	51	29.31%	0.0001
	No	163	67.08%	89	51.15%	
	Declined	17	7.00%	34	19.54%	
Physical therapy (Y/N)	Yes	80	32.92%	85	48.85%	0.0015
	No	163	67.08%	89	51.15%	

Vitamin D Level

Vitamin D levels were recorded in only 73% (n=177) of the patients. The average level of vitamin D was 37.54 ng/ml (SD=15.99). Of those 177 patients, levels below 30 ng/ml were seen in 61 patients (35%). For those 61 patients, only 57% (n=35) were on vitamin D supplementation. 

Part 2

A survey was completed by a total of 43 providers in the IM and FM. About half of providers saw between 1-10 patients annually for falls, and 35% saw between 11-20 patients annually for the same. About 70% of providers believed vitamin D to be deficient at levels <30 ng/ml. About 61% of providers believed that vitamin D deficiency was a significant cause of falls in the elderly. Most believed that vitamin D supplementation was helpful in the prevention of falls, but less prescribed it for fall prevention (Table 2).

**Table 2:** The providers' view on vitamin D deficiency as a cause of falls SD: standard deviation This shows that providers believe vitamin D deficiency is an important cause of falls, and that vitamin D supplementation should be added for the prevention of the same.

How important do you think vitamin D supplementation is for the prevention of falls?	Do you think that vitamin D deficiency is a significant cause of falls?					p-value
		Yes		No		
		Count	Proportion	Count	Proportion	
	Important	7	26.92%	1	6.25%	0.009
	Somewhat important	18	69.23%	8	50.00%	
	Neither important nor unimportant	1	3.85%	5	31.25%	
	Somewhat unimportant	0	0.00%	1	6.25%	
	Unimportant	0	0.00%	1	6.25%	
		Mean	SD	Mean	SD	p-value
		4.23	0.51	3.44	0.96	0.006

Many providers stated that they sometimes or frequently refer patients that had fallen to HH or PT to reduce the risk of a fall. Most providers think that polypharmacy may cause a fall and educate their patients on potential side effects. Very few providers believe that calcium supplementation reduces falls and was infrequently prescribed (Table 3).

**Table 3:** Providers' view on calcium This shows that providers believe calcium was not important in fall prevention, and they did not advise patients to add calcium to their diet.

How often do you educate patients on adding calcium in their diets as part of fall prevention?		How important do you think calcium supplementation is for prevention of falls?					
		Important	Somewhat important	Neither important nor unimportant	Somewhat unimportant	Unimportant	
		Count	Count	Count	Count	Count	p-value
	Very Frequently	1	0	0	0	0	<0.001
	Frequently	1	6	1	0	1	
	Sometimes	2	9	1	0	0	
	Infrequently	1	1	3	1	2	
	Very infrequently	1	0	7	0	5	

A presentation was given at grand rounds discussing a multidisciplinary treatment approach for patients that had fallen. During the presentation, an FM physician, an IM physician, a physical therapist, and an HH specialist discussed ways to evaluate elderly patients who had fallen. A patient care protocol was discussed, which included looking at medications, evaluating patient's balance, checking vitamin D deficiency, and appropriate referrals.

Part 3

Following post-education and the development of patient care protocol, most providers believed vitamin D to be deficient between 20-30 ng/ml, and these results were consistent with the levels reported in the pre-intervention survey (Table 1). The majority believed that vitamin D supplementation was important to minimize falls.

Most providers responded that they monitored vitamin D levels yearly for those who have fallen. Providers agreed that PT or HH was important for patients who had fallen, and most of them frequently referred them. About 95% of providers answered that polypharmacy was common among those who had fallen.

Most providers believed that vitamin D supplementation was important for the prevention of falls and prescribed it for their patients (p = 0.0028) (Table 4).

**Table 4:** Vitamin D importance Providers who believed that vitamin D was a significant cause of falls would prescribe vitamin D to their patients.

How often do you prescribe vitamin D?		Vitamin D Importance										p-value
		Important		Somewhat Important		Neither important nor unimportant		Somewhat unimportant		Unimportant		
		Count	Proportion	Count	Proportion	Count	Proportion	Count	Proportion	Count	Proportion	
	Very frequently	5	36%	1	6%	0	0%	0	0%	0	0%	0.0028
	Frequently	7	50%	6	33%	0	0%	0	0%	0	0%	
	Sometimes	1	7%	4	22%	3	60%	1	50%	0	0%	
	Infrequently	0	0%	5	28%	0	0%	1	50%	1	33%	
	Very Infrequently	1	7%	2	11%	2	40%	0	0%	2	67%	

Most providers did not think that calcium supplementation was important for prevention of falls, and they did not prescribe it for their patients (p = 0.0001) (Table 5).

**Table 5:** Calcium importance Providers did not consider calcium as an important cause of falls and did not educate patients about adding calcium to their diets.

How often do you prescribe calcium?		Calcium Importance										p-value
		Important		Somewhat Important		Neither important nor unimportant		Somewhat unimportant		Unimportant		
		Count	Proportion	Count	Proportion	Count	Proportion	Count	Proportion	Count	Proportion	
	Very frequently	8	73%	1	7%	0	0%	0	0%	0	0%	0.0001
	Frequently	0	0%	5	36%	0	0%	0	0%	0	0%	
	Sometimes	2	18%	4	29%	2	25%	0	0%	1	17%	
	Infrequently	1	9%	3	21%	3	38%	2	67%	1	17%	
	Very Infrequently	0	0%	1	7%	3	38%	1	33%	4	67%	

After the completion of the education period, 176 patients were seen over a period of about six months. Most patients that were seen in the clinic who had fallen were female (72%). About 60% of falls were deemed to be mechanical in origin. Almost all (95%) of patients seen were on at least four medications. About 30% of patients had low vitamin D levels, but only 61% of those patients with low levels were being treated with vitamin D. HH was offered to patients about 8% of the time, and PT was offered about 50% of the time. In the IM department, the number of patients that were recommended for PT went from about 34% to about 55% (p = <0.0001).

## Discussion

Healthcare providers entering the outpatient primary care environment will have many elderly patients who have either fallen or at risk of falling. Therefore, a consistent diagnosing and treatment approach for fall victims is important for good patient care. Two years of past patient records of fall victims that saw either a family or internal medicine doctor were reviewed to establish the current medical treatment approach. Treatment approaches used were inconsistent between providers. Some patients were placed on supplements, some obtained medication usage counseling, and some obtained referrals for either PT or HH. The diagnosis of why someone fell was not consistently documented. Therefore, the hospital implemented a patient care protocol for patients that had fallen.

Pre-intervention

Most elderly patients who are fall victims should be tested to measure if the amount of vitamin D in their system is adequate and receive either PT or HH for strengthening and balance. However, patient records revealed inconsistent treatment practices between providers:

- Only 73% of patients had their vitamin D level measured.

- Among the patients whose vitamin D level had been measured and found to be below the 30 ng/ml requirement, only 57% were placed on supplements.

- Only 45% of patients were recommended to receive either PT (33%) or HH (12%).

According to the survey results, most providers believed that PT/HH was helpful in managing patients with fall risk. Only 45% of patients were recommended due to the knowledge the provider has that some of their patients were unwilling to participate in therapy. Also, therapy follow-up would require the provider to spend time following the patient through the therapy process, taking time away from other patient care.

Most providers interviewed in the survey believe that vitamin D deficiency increases the risk of falling in elderly patients and that supplements should be taken (Table 2). This is also inconsistent with the medical records that were reviewed (73% recommendation). The inconsistency can be explained by noting that some of the patients were not seen by the doctors who were interviewed. Also, a provider may believe that vitamin D supplementation is important, but not always act on it, nor always document it in the chart due to time constraints.

Both vitamin D and calcium work together in the body for bone health. Vitamin D helps in calcium absorption and builds skeletal health by bone mineralization. Thus, current recommendations from the American Geriatrics Society (AGS) have patients 65 years of age and older taking both vitamin D and calcium to help prevent osteoporosis and falls [9]. However, those who participated in the survey did not believe calcium was important in fall prevention, and did not advise patients to add calcium into their diet (Table 3). The AGS also states there is insufficient data to support the recommendation of increased vitamin D supplementation without calcium [9]. It is unclear why providers chose to ignore the role of calcium supplements in strengthening bone structure. They may think patients already ingest enough calcium in their diets. Or, they may be concerned about possible side effects of calcium supplementation including nephrolithiasis and cardiovascular disease that some studies have shown with calcium supplementation alone [2].

Education

A multidisciplinary team consisting of an FM physician, an IM physician, a physical therapist, and an HH nurse presented at grand rounds. During this grand rounds, information on Vitamin D, calcium, and referrals was discussed. The IM physician discussed a patient, who had fallen, and all the medications that she was on. The risk of polypharmacy was emphasized. A physical therapist spoke of various treatment modalities that are helpful for patients with balance and deconditioning. The HH nurse showed an example of risk to a patient trying to navigate their house.

The grand rounds event was well attended by a variety of healthcare professionals (10% of participants, 58% of residents from FM; 50% of participants, 66% of residents from IM).

Post-intervention

A year after the education was completed, post-intervention started. The EMR was surveyed to observe the changes in patient care. Of the 176 patients seen by a provider after a fall, most patients were female (73%). Causation of falls was 59% mechanical, 22% risk of falls (history of falls, poor mobility or balance, etc.), 12.5% unknown, and 6% cardiovascular/neurological. Polypharmacy was evident again (as defined as >4 medications/supplements) in 95% of patients.

- The percentage of patients that had their vitamin D levels drawn was 77.8% (137 patients). This was a slight improvement from the pre-intervention results of 73%.

- Of patients whose vitamin D levels were low, 60% were on supplements (n=25), a small improvement from those prescribed supplements before training was done (57%).

- The number of those referred to HH care remained about the same.

- There was a significant increase (see Table 1) in patients referred to PT (48.8%) after provider education than prior (33.0%). The increase in referrals came from the internal medicine providers (Tables 6 and 7). This may be attributed to a relatively high attendance of IM providers at the grand rounds as compared to a relative few attendees from FM.

**Table 6:** Family medicine pre-intervention vs. post-intervention data vs: versus; n: number; CV: cardiovascular; neuro: neurological; Y: yes; N: no

Family practice pre-intervention vs. post-intervention						
		Pre-intervention (n = 73)		Post-intervention (n = 43)		p-value
		Count	Proportion	Count	Proportion	
Gender	Female	43	58.11%	31	72.09%	0.1889
	Male	31	41.89%	21	27.91%	
Cause of fall	Mechanical	58	78.38%	27	62.79%	0.0020
	Multifactorial	3	4.05%	0	0.00%	
	Drugs	3	4.05%	0	0.00%	
	CV/neuro	7	9.46%	4	9.30%	
	Risk of falls	3	4.05%	7	11.63%	
	Blind	0	0.00%	0	0.00%	
	Unknown	0	0.00%	5	16.28%	
Polypharmacy	Yes	67	90.54%	41	95.35%	0.4825
	No	7	9.46%	2	4.65%	
Low vitamin D	Yes	14	37.84%	7	35.00%	1.0000
	No	23	62.16%	13	65.00%	
Vitamin D supplements	Yes	32	43.24%	15	34.88%	0.4879
	No	42	56.76%	28	65.12%	
Vitamin D supplements if low vitamin D	Yes	9	64.29%	4	57.14%	1.0000
	No	5	35.71%	3	42.86%	
Low calcium	Yes	0	0.00%	0	0.00%	
	No	117	100.00%	117	100.00%	
Calcium supplements	Yes	19	25.68%	11	25.58%	1.0000
	No	55	74.32%	32	74.42%	
Home health	Yes	6	8.11%	1	2.38%	0.3489
	No	64	86.49%	40	95.24%	
	Declined	4	5.41%	1	2.38%	
Home health (Y/N)	Yes	10	13.51%	2	4.76%	0.2419
	No	64	86.49%	40	95.24%	
Physical therapy	Yes	18	23.43%	7	17.07%	0.5538
	No	51	68.92%	30	73.17%	
	Declined	5	6.76%	4	9.76%	
Physical therapy (Y/N)	Yes	23	31.08%	11	26.83%	0.7908
	No	51	68.92%	30	73.17%	

**Table 7:** Internal medicine pre-intervention vs. post-intervention data vs: versus; n: number; CV: cardiovascular; neuro: neurological; Y: yes; N: no

Internal medicine pre-intervention vs .post-intervention						
		Pre-intervention (n = 169)		Post-intervention (n = 133)		p-value
		Count	Proportion	Count	Proportion	
Gender	Female	115	68.05%	97	72.93%	0.4268
	Male	54	31.95%	36	37.01%	
Cause of fall	Mechanical	119	70.41%	77	57.89%	0.0005
	Multifactorial	5	2.96%	0	0.00%	
	Drugs	3	1.78%	0	0.00%	
	CV/neuro	17	10.06%	7	5.26%	
	Risk of falls	5	2.96%	32	24.06%	
	Blind	1	0.59%	0	0.00%	
	Unknown	19	11.24%	17	12.78%	
Polypharmacy	Yes	150	88.76%	126	94.74%	0.1026
	No	19	11.24%	7	5.26%	
Low vitamin D	Yes	47	33.57%	34	29.06%	0.5219
	No	93	66.43%	83	70.94%	
Vitamin D supplements	Yes	92	54.44%	77	57.89%	0.6284
	No	77	45.56%	56	42.11%	
Vitamin D supplements if low vitamin D	Yes	26	55.32%	21	61.76%	0.7248
	No	21	44.68%	13	38.24%	
Low calcium	Yes	4	2.37%	4	3.01%	0.7346
	No	165	97.63%	129	96.99%	
Calcium supplements	Yes	36	21.30%	33	24.81%	0.5597
	No	113	78.70%	100	75.19%	
Home health	Yes	15	8.88%	12	9.02%	0.8326
	No	151	89.35%	120	90.23%	
	Declined	3	1.78%	1	0.75%	
Home health (Y/N)	Yes	18	10.65%	13	9.77%	0.9536
	No	151	89.35%	120	90.23%	
Physical therapy	Yes	45	26.63%	44	33.08%	< 0.0001
	No	112	66.27%	59	44.36%	
	Declined	12	7.10%	30	22.56%	
Physical therapy (Y/N)	Yes	57	33.73%	74	55.64%	0.0002
	No	112	66.27%	59	44.36%	

The post-education survey was completed by 42 providers from FM and IM. About 95% of respondents considered vitamin D levels to be deficient at <20 ng/ml or <30 ng/ml. Two-thirds of respondents believed vitamin D deficiency to be a significant cause of falls. The providers who believe that vitamin D deficiency is a significant cause of falls prescribed vitamin D to their patients (p=0.0028, Table 4). Providers also did not consider calcium to be important for fall prevention and therefore, did not educate patients about adding calcium into their diets (p=0.0001, Table 5). About 80% of providers answered that they frequently referred patients to either HH or PT. All providers answered that either they believed that polypharmacy was very frequently (57%), frequently (38%), or sometimes (5%) was a cause of falls. All providers answered that they educated patients on medications and side effects very frequently (40.5%), frequently (50%), or sometimes (9.5%).

The study had some significant findings. Most providers only prescribe vitamin D supplements when necessary to raise a patient’s test results to the standard. However, supplements were not prescribed as a form of fall prevention for those who had acceptable levels of vitamin D present in their bloodstream. Conversely, most providers did not think calcium supplementation reduced the risk of fractures when people fell and did not educate their patients on the importance of calcium in their diets. IM providers increased the number of referrals to PT after the education while FM providers, who mostly absent from the session, did not. Otherwise, there were no statistically significant changes in patient care between pre-intervention and post-intervention surveys for FM and IM providers.

There may be an opportunity for a quality improvement project to address education on referrals of fall victims to PT within the FM program. A future study could review the patient medical records to determine the usefulness of PT in reducing falls.

## Conclusions

Elderly people are more prone to fall as they age. This can increase a person’s morbidity and mortality rate. It is important to have an established process in place when caring for patients who have fallen. This study attempted to establish a process for evaluating fall victims. Vitamin D with calcium supplementation has been shown to be significant in decreasing fall risk for patients. PT and HH are important adjuncts for decreasing falls. Further research and education are required to unify how providers care for patients that have fallen.

## Appendices

Supplementary Text S1:

Survey given to family medicine and internal medicine providers before intervention occurred.

Fall Survey

1.       Approximately how many unique patients do you see for falls annually?

a.       0

b.       1-10

c.       11-20

d.       21-30

e.       31-40

f.        40+

2.       How confident do you feel about taking care of patients with falls?

a.       Confident

b.       Somewhat confident

c.       Neither confident nor unconfident

d.       Somewhat unconfident

e.       Unconfident

3.       When a patient presents who has fallen, do you have a specific way to evaluate that patient?

a.       Yes

b.       No

4.       At what level would you consider vitamin D to be deficient?

a.       <20 ng/ml

b.       <30 ng/ml

c.       <40 ng/ml

d.       <50 ng/ml

5.       Do you think that vitamin D deficiency is a significant cause of falls?

a.       Yes

b.       No

6.       How important do you think vitamin D supplementation is for the prevention of falls?

a.       Important

b.       Somewhat important

c.       Neither important nor unimportant

d.       Somewhat unimportant

e.       Unimportant

7.       How often do you prescribe vitamin D for fall prevention?

a.       Very frequently

b.       Frequently

c.       Sometimes

d.       Infrequently

e.       Very infrequently

8.       How often do you monitor vitamin D levels for those at risk for falls or for those who have fallen?

a.       Every few years

b.       Yearly

c.       Monthly

d.       Weekly

e.       Once

f.        Never

9.       How often do you refer patients who have fallen to home health or physical therapy?

a.       Very frequently

b.       Frequently

c.       Sometimes

d.       Infrequently

e.       Very infrequently

10.   Have you decreased referring to home-health due to the requirement of having a recent face-to-face visit with the patient?

a.       Yes

b.       No

11.   How important do you think physical therapy or home health therapy is to helping patients with fall risk?

a.       Important

b.       Somewhat important

c.       Neither important nor unimportant

d.       Somewhat unimportant

e.       Unimportant

12.   How often do you assess gait as part of your visit for patients who have fallen?

a.       Very frequently

b.       Frequently

c.       Sometimes

d.       Infrequently

e.       Very infrequently

13.   How often do you have patients who are in wheelchairs get out of their chair when they are seen after a fall?

a.       Very frequently

b.       Frequently

c.       Sometimes

d.       Infrequently

e.       Very infrequently

14.   How often do you prescribe mobility devices to patients who have fallen?

a.       Very frequently

b.       Frequently

c.       Sometimes

d.       Infrequently

e.       Very infrequently

15.   How often do you assess your patients’ current mobility devices?

a.       Very frequently

b.       Frequently

c.       Sometimes

d.       Infrequently

e.       Very infrequently

16.   How often do you think that polypharmacy is a cause of falls?

a.       Very frequently

b.       Frequently

c.       Sometimes

d.       Infrequently

e.       Very infrequently

17.   How often do you educate your patients on medications and side effects?

a.       Very frequently

b.       Frequently

c.       Sometimes

d.       Infrequently

e.       Very infrequently

18.   How many medications do you define as polypharmacy?

a.       4

b.       5-10

c.       10+

19.   Would you consider supplements as a part of medications to be counted towards polypharmacy?

a.       Yes

b.       No

20.   How important do you think calcium supplementation is for prevention of falls?

a.       Important

b.       Somewhat important

c.       Neither important nor unimportant

d.       Somewhat unimportant

e.       Unimportant

21.   How often do you educate patients on adding calcium into their diets as part of fall prevention?

a.       Very frequently

b.       Frequently

c.       Sometimes

d.       Infrequently

e.       Very infrequently

22.   How often do you document if patients are frail?

a.       Very frequently

b.       Frequently

c.       Sometimes

d.       Infrequently

e.       Very infrequently

Supplementary Text S2:

Survey given to family medicine and internal medicine providers after intervention occurred.

Post Education Fall Survey

1.   At what level would you consider vitamin D to be deficient?

a.       <20 ng/ml

b.       <30 ng/ml

c.       <40 ng/ml

d.       <50 ng/ml

2.   Do you think that vitamin D deficiency is a significant cause of falls?

a.       Yes

b.       No

3.   How important do you think vitamin D supplementation is for the prevention of falls?

a.       Important

b.       Somewhat important

c.       Neither important nor unimportant

d.       Somewhat unimportant

e.       Unimportant

4.   How often do you prescribe vitamin D for fall prevention?

a.       Very frequently

b.       Frequently

c.       Sometimes

d.       Infrequently

e.       Very infrequently

5.   How often do you monitor vitamin D levels for those at risk for falls or for those who have fallen?

a.       Every few years

b.       Yearly

c.       Monthly

d.       Weekly

e.       Once

f.        Never

6.   How often do you refer patients who have fallen to home health or physical therapy?

a.       Very frequently

b.       Frequently

c.       Sometimes

d.       Infrequently

e.       Very infrequently

7.   How important do you think physical therapy or home health therapy is to helping patients with fall risk?

a.       Important

b.       Somewhat important

c.       Neither important nor unimportant

d.       Somewhat unimportant

e.       Unimportant

8.   How often do you think that polypharmacy is a cause of falls?

a.       Very frequently

b.       Frequently

c.       Sometimes

d.       Infrequently

e.       Very infrequently

9.   How often do you educate your patients on medications and side effects?

a.       Very frequently

b.       Frequently

c.       Sometimes

d.       Infrequently

e.       Very infrequently

10.   How important do you think calcium supplementation is for prevention of falls?

a.       Important

b.       Somewhat important

c.       Neither important nor unimportant

d.       Somewhat unimportant

e.       Unimportant

11.   How often do you educate patients on adding calcium into their diets as part of fall prevention?

a.       Very frequently

b.       Frequently

c.       Sometimes

d.       Infrequently

e.       Very infrequently
